# Association Between Depression and Lung Cancer Risk Among Postmenopausal Women

**DOI:** 10.1002/cam4.70695

**Published:** 2025-03-02

**Authors:** Yuanyuan La, Su Yon Jung, Xiaoyun Liang, Michelle J. Naughton, Michael Hendryx, Juhua Luo

**Affiliations:** ^1^ School of Government Beijing Normal University Beijing China; ^2^ Translational Sciences Section, School of Nursing Department of Epidemiology, Fielding School of Public Health, Jonsson Comprehensive Cancer Center, University of California Los Angeles California USA; ^3^ Division of Cancer Prevention and Control, Department of Internal Medicine The Ohio State University Columbus Ohio USA; ^4^ Department of Environmental and Occupational Health, School of Public Health Indiana University Bloomington Bloomington Indiana USA; ^5^ Department of Epidemiology and Biostatistics School of Public Health, Indiana University Bloomington Bloomington Indiana USA

**Keywords:** depression, incidence, lung cancer, postmenopausal women

## Abstract

**Background:**

In recent years, the association between depression and various chronic diseases has attracted widespread attention. However, the effect of depression on lung cancer incidence has not been well studied. This study aimed to explore whether depression increases the incidence of lung cancer and to analyze the mediating and moderating roles of smoking in this relationship.

**Methods:**

This study used large‐scale longitudinal data sourced from the Women's Health Initiative, encompassing 123,961 postmenopausal women. Depressive symptoms were measured using the 8‐item Burnam regression algorithm with a cut‐point of 0.06, and depression was defined as either depressive symptoms or antidepressant use at baseline. The relationship between depression and lung cancer incidence was examined using a multivariate Cox proportional hazards regression model. A four‐way decomposition causal mediation approach was employed to investigate the potential mediating and moderating effects of smoking.

**Results:**

After a mean follow‐up of 17.6 years, 3434 cases of lung cancer were identified. The incidence rate of lung cancer was higher among individuals with depression compared to those without (HR: 1.15, 95% CI: 1.05–1.26). Cigarette smoking partially mediated the relationship between depression and lung cancer incidence, explaining about 27% of the association effect.

**Conclusions:**

This study identified a significant association between depression and lung cancer incidence, and smoking partially mediates this relationship. This highlights that managing depression may play a key role in reducing lung cancer risk and decreasing tobacco use. Psychological support should be integrated with traditional smoking cessation programs for lung cancer prevention.

## 1 Introduction

In 2022, the Global Cancer Observatory reported that lung cancer held the highest position both in terms of new cancer cases (12.4%) and cancer‐specific deaths, contributing to nearly 1.8 million (18%) of all cancer‐related deaths worldwide [1]. While smoking remains the primary risk factor contributing to lung cancer, other risk factors are increasingly recognized as key contributors to the development of lung cancer in women [2]. Besides, females who never smoked developed lung cancer at higher rates than their male counterparts [3, 4]. Therefore, identifying other modifiable and non‐modifiable risk factors for lung cancer in women has critical implications for reducing its incidence and mortality.

Depression ranks among the most prevalent mental health disorders and has long been assumed to elevate the cancer risk [5, 6]. There have been meta‐analyses on depression and overall cancer incidence and on specific cancer types such as breast cancer and ovarian cancer, but meta‐analysis results for lung cancer have been inconsistent in these studies. For instance, some meta‐analyses have found an association between depression and a higher incidence of lung cancer [6, 7] while others showed no association [8]. The reasons for the different results may lie in the selection of different confounding variables, variations in sample sizes, and discrepancies in the measurement of depression. Therefore, additional replication studies are needed with large sample sizes, better depression assessment, and a comprehensive selection of potential confounders.

The prevalence of depression is twice as high in women compared to men [9], and is higher among postmenopausal women than pre‐menopausal women [10]. Additionally, research has suggested that depressive symptoms in postmenopausal women may be more severe than in premenopausal women, with early postmenopausal women being 2–4 times more likely to experience a major depressive episode than premenopausal women [11]. Women will spend approximately one‐third of their lives postmenopausal, making it crucial to study the impact of depression on the health of postmenopausal women. However, there is limited epidemiological research exploring the association between depression and lung cancer in women, with no research specifically focused on postmenopausal women. A study conducted on a nationwide cohort in Taiwan explored the link between depression and lung cancer incidence, revealing that women with depression faced a higher risk of lung cancer [12]. However, this study did not adjust for smoking and drinking factors. Another prospective study based on the Nurses' Health Study (NHS) for over 24 years cohort found that women with the most severe depressive symptoms faced a significantly increased risk of developing lung cancer as opposed to those with the lowest [13]. However, this study did not take into account the impact of antidepressant use on lung cancer. Of note, those studies have not examined study participants by menopause status. Therefore, we focused on postmenopausal women, a highly vulnerable population to depression and lung cancer by using the Women's Health Initiative (WHI), a larger prospective cohort study with 30 years of follow‐up data, primarily focused on postmenopausal women's health.

Furthermore, smoking as a leading risk factor for developing lung cancer is strongly associated with depression [14, 15]. However, how these factors in combination influence lung cancer risk is unclear. Previous mechanistic studies have suggested that depression could result in changes in the immune system, which could subsequently affect cellular mutation, viral oncogenes, cell proliferation, and DNA repair [16]. The effects of smoking on lung cancer can also result from the influence of specific carcinogens and mutagens in cancer‐related genes, including those associated with DNA repair [17]. These biological pathways may have a synergy effect on increased lung cancer risk among people with depression who smoke. Thus, smoking likely moderates the link between depression and lung cancer. Alternatively, individuals with depression may turn to smoking as a means of alleviating their symptoms [14, 15], which in turn increases the risk of lung cancer. Thus, smoking could play a mediating role in the depression –lung cancer relationship. Findings from the existing epidemiological studies on this topic are inconsistent. One study based on the UK Biobank cohort found that smoking served as a significant mediator and moderator in the depression–lung cancer association [18]. Another study with the NHS found that this association was only partially mediated but not moderated by smoking in the NHS [13]. Thus, a comprehensive investigation into the potential mediating and moderating role of smoking is needed.

## 2 Methods

### 2.1 Data and Participants

Data are from the WHI Observational Study (WHI‐OS) and three Clinical Trials (WHI‐CTs), which are two study arms, providing pivotal information for research on postmenopausal women's health. The WHI study, initiated in the early 1990s, recruited 161,808 postmenopausal women from 40 clinical centers across the country between 1993 and 1998. All WHI participants provided informed consent in writing and were followed prospectively [19, 20].

**FIGURE 1:**
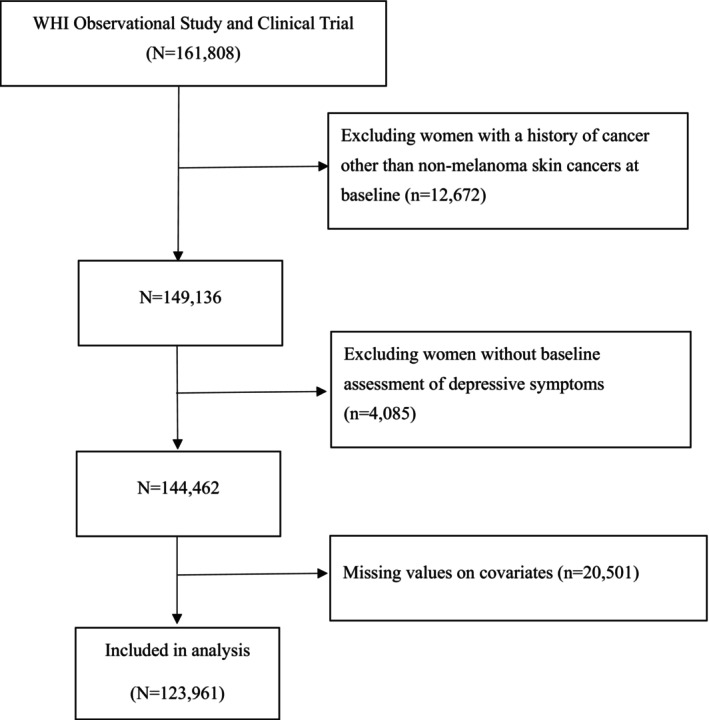
Flow diagram of the participants included in the analysis.

The participants listed below were removed from the initial cohort for this study (Figure 1): 12,672 women with a history of cancer other than nonmelanoma skin cancers at baseline, 4085 women without baseline assessment of depressive symptoms, and 20,501 women with missing data on covariates (including age, race, education, diet quality score, BMI, physical activity, smoking status, pack‐years of smoking, history of emphysema, and family history of cancer). After applying the exclusion criteria, 123,961 women remained for further analysis.

### 2.2 Exposure Measurement

In the WHI, depressive symptoms were assessed at baseline for all WHI‐OS and WHI‐CT participants (CT: *n* = 68,132, OS: *n* = 93,676) using the Burnam 8‐item scale [20, 21]. This scale is comprised of six items from the Center for Epidemiologic Studies‐Depression (CES‐D) scale and two items from the Diagnostic Interview Schedule (DIS). The Burnam score, which ranges from 0 to 1, signifies more severe depressive symptoms with a higher score [21, 22]. Following prior research, we converted this continuous variable into a dichotomous variable using the pre‐established threshold, with WHI participants scoring 0.06 or above classified as depressed [23, 24]. The Burnam scale shows a 74% sensitivity and an 87% specificity in comparison to clinical diagnoses of depression [25].

For antidepressant use, WHI participants were asked to bring containers of both prescription and over‐the‐counter medication to their baseline WHI appointment. The medication details were then recorded into a medication database obtained from the Master Drug Database. The National Drug Code associated with Antidepressant Medications Management was used to assess whether the medication was categorized as an antidepressant [26]. Based on this information, women were categorized as either antidepressant users or nonusers.

A joint variable called “depression” was also used to account for the close relationship between depressive symptoms and antidepressant use. In this study, “depressed” referred to women who either had depressive symptoms over the cutoff score or used antidepressants at baseline, while “non‐depressed” referred to those with neither factor.

### 2.3 Outcomes Measurement

The incidence of lung cancer was the primary outcome of interest. Lung cancer diagnosis was first made based on self‐reported questionnaires, followed by confirmation through pathology report reviews, and finally centrally adjudicated. Survival time was right‐censored, calculated from the baseline assessment until death, lung cancer diagnosis, loss to follow‐up, or end of follow‐up (February 2023), whichever occurred first. The average follow‐up time of this study is 17.6 years.

In WHI, information about lung cancer tumor characteristics was coded by trained professionals according to the Surveillance, Epidemiology, and End Results (SEER) program, utilizing the second edition of the International Classification of Disease on Oncology (ICDO) [27]. Specifically, lung cancer histology was defined as small cell lung cancer (*n* = 241), squamous cell carcinoma (*n* = 357), adenocarcinoma (*n* = 1367), and other non‐small cell lung cancer (*n =* 793). Lung cancer stage was defined as localized (*n* = 820), regional (*n* = 642), distant (*n* = 1046), and unknown (*n* = 244). Lung cancer grade was defined as well differentiated (*n* = 299), moderately differentiated (*n* = 519), poorly differentiated (*n* = 484), anaplastic (*n* = 98), and unknown (*n* = 1358).

### 2.4 Mediator/Moderator: Smoking

In our study, we used the continuous pack‐years of smoking as a mediator/moderator. Cigarette smoking information was collected at baseline. Pack‐years of smoking were a calculated variable that considers the number of cigarettes smoked daily on average and the total years of smoking.

### 2.5 Covariates

Based on previous studies [13, 18] and risk factors for lung cancer published by the American Cancer Society, we selected potential confounders including age (continuous), race (American Indian/Alaskan native, Native Hawaiian/Other Pacific Islander, White, Black, Asian, more than one race, unknown/not reported), education (high school or less, some college or technical training, college degree or higher), diet quality score as a continuous variable (HEI‐2015), BMI (< 25, 25–< 30, ≥ 30), physical activity as a continuous variable (METs/week), pack‐years of smoking (never smoker, < 5, 5‐ < 20, ≥ 20), history of emphysema (yes/no), family history of cancer (yes/no), participation study group (OS or CTs).

### 2.6 Statistical Analysis

We described continuous variables using means ± standard deviations, and categorical variables were represented as proportions. *t*‐tests were utilized to assess differences by depression status (yes or no) in continuous variables, while Chi‐squared tests were applied to categorical variables. Statistical significance was evaluated at *p* < 0.05.

We used Cox proportional hazard models to evaluate the relationship between depression and lung cancer risk during the follow‐up period. The model was first adjusted only for age. In the second model, additional covariates (BMI, diet quality score, physical activity, history of lung cancer, and WHI participation in study groups) were added as covariates to the first model. A third model was additionally adjusted for pack‐years of smoking. Additionally, we assessed this association specifically among those who never smoked.

To further explore how smoking influences the relationship between depression and incident lung cancer, we performed a causal mediation analysis using a framework of a Cox proportional hazards model. This analysis was designed to evaluate the mediation and interaction effects through continuous pack‐years of smoking. We used the four‐way counterfactual approach developed by Vanderweele and implemented it using the user‐written *med4way* command in Stata [28]. Essentially, the method decomposes the total effect (TE) of depression on lung cancer incidence into four distinct parts: the controlled direct effect (CDE), which results from neither mediation nor interaction; the reference interaction (INTref), driven solely by interaction; the mediated interaction (INTmed), with results from both mediation and interaction; and the pure indirect effect (PIE), driven only by mediation (Figure S1).

Two sensitivity analyses were conducted to assess the reliability of our findings. First, given the higher sensitivity but slightly lower specificity of the 0.009 cut‐point in comparison to the 0.06 threshold for both recent and lifetime prevalence of depressive symptoms [29], we re‐categorized depressive symptoms into categories of ‘no’ or ‘yes’ using a threshold of 0.009 to repeat the main analysis throughout the follow‐up period and to test the effect of smoking's mediation and interaction. Second, we analyzed the effects of depression on lung cancer incidence owing to mediation and interaction with pack‐years of smoking at the 75th percentile. We conducted all analyses utilizing Stata version 16.0.

## 3 Results

Among the 123,961 postmenopausal women, the prevalence of women who have been depressed based on the depressive symptom score or antidepressant use at baseline was 14.15%. Women with depression were found to be younger, have lower levels of education, be physically inactive, have higher BMI, have higher pack‐years of smoking, and be in the WHI‐OS compared to those without depression. They also tended to have lower diet quality scores, a higher prevalence of emphysema history, higher rates of family history of cancer, and were less likely to have never smoked (Table 1).

**TABLE 1:** Baseline characteristics of participants by depression in the WHI (*n* = 123,961).

Variable	Depression at baseline		
	Yes	No	*p*
Total number of women	17,544	106,417	
Age (mean ± SD, years)	61.85 ± 7.24	63.39 ± 7.15	< 0.001
Race (%)			< 0.001
American Indian/Alaskan native	0.42	0.28	
Asian	1.44	2.93	
Native Hawaiian/Other Pacific islander	0.06	0.09	
Black	8.78	8.08	
White	84.91	86.02	
More than one race	1.40	1.13	
Unknown/not reported	2.98	1.47	
Education (%)			< 0.001
High school or less	26.19	21.30	
Some college or technical training	39.92	37.48	
College degree or higher	33.90	41.22	
Body mass index (kg/m^2^, %)			< 0.001
< 25	29.72	36.70	
25–< 30	33.68	34.84	
≥ 30	36.61	28.46	
Physical activity (mean ± SD, METs/week)	10.17 ± 12.50	12.85 ± 13.83	< 0.001
Smoking (%)			< 0.001
Never smoked	47.83	53.11	
Former smoker	41.93	40.57	
Current smoker	10.24	6.32	
Pack‐years of smoking (%)			< 0.001
Never smoker	47.83	53.11	
< 5	15.00	14.51	
5–< 20	14.19	14.45	
≥ 20	22.98	17.94	
Group (%)			< 0.001
CTs	40.09	42.27	
OS	59.91	57.73	
HRT participant			0.01
Yes	16.29	17.08	
No	83.71	82.92	
DM participant			< 0.001
Yes	28.71	30.05	
No	71.29	69.95	
CAD participant			< 0.001
Yes	21.11	22.89	
No	78.89	77.11	
Diet quality score (mean ± SD)	63.18 ± 10.72	65.57 ± 10.30	< 0.001
History of emphysema (%)	6.05	3.11	< 0.001
Family history of cancer (%)	67.08	66.17	0.018

Abbreviations: CAD, calcium and vitamin D; CT: clinical trials; DM: dietary modification; HRT: hormone replacement therapy; OS: observational study; SD: standard deviation; WHI: women's health initiative.

Over an average follow‐up of 17.6 years among 123,961 postmenopausal women, 3434 cases of lung cancer were identified. Compared to women without depression, those with depression showed a higher risk of lung cancer in the age‐adjusted model, the sociodemographic‐adjusted model, and the fully adjusted models (HR: 1.37, 95% CI: 1.25–1.50; HR: 1.25, 95% CI: 1.14–1.37; HR: 1.15, 95% CI: 1.05–1.26), respectively. The same significant association was observed in women with depressive symptoms versus those without across age, sociodemographic, and fully adjusted models. However, in women who had used antidepressants, this association was not significant after further adjustment for pack‐years of smoking (HR: 1.07, 95% CI: 0.93–1.24) (Table 2).

**TABLE 2:** Hazard ratios (HRs) and 95% confidence intervals (CIs) for lung cancer incidence in relation to depression at baseline (*n* = 123,961).

	*n*	Number of cases	Age‐adjusted model^a^	Sociodemographic‐adjusted model^b^	Fully‐adjusted models^c^
			HR (95% CI)	HR (95% CI)	HR (95% CI)
Depression					
No	106,417	2885	1	1	1
Yes	17,544	549	1.37 (1.25–1.50)	1.25 (1.14–1.37)	1.15 (1.05–1.26)
Depressive symptoms					
No	110,811	3028	1	1	1
Yes	13,150	406	1.36 (1.22–1.51)	1.21 (1.10–1.35)	1.14 (1.03–1.27)
Antidepressant use					
No	117,820	3242	1	1	1
Yes	6141	192	1.29 (1.12–1.50)	1.23 (1.07–1.43)	1.07 (0.93–1.24)

*Note:* Depression was defined as women with either depressive symptoms or antidepressant use at baseline.

^a^ Model 1 adjusted for age (continuous).

^b^ Model 2 adjusted for Model 1 + education (high school or less, some college or technical training, college degree or higher), race (American Indian or Alaskan Native, Asian, Native Hawaiian/Other Pacific Islander, Black, White, More than one race, Unknown/Not reported), history of emphysema (yes/no), family history of cancer (yes/no), body mass index (< 25, 25–< 30, ≥ 30), diet quality score (continuous), physical activity (continuous), study group(CTs, OS), HRT trial (yes/no), DM trial (yes/no), CAD trial (yes/no).

^c^ Model 3 adjusted for Model 2 + pack‐years of smoking (never smoker, < 5, 5–< 20, ≥ 20).

In the analysis of lung tumor histology, stage, and grade, we observed a significant association for small cell lung cancer after adjustment for potential confounders (HR: 1.44, 95% CI: 1.05–1.96). Besides, depression was linked to an increased risk of regional lung cancer (HR: 1.35, 95% CI: 1.10–1.66) and well‐differentiated lung cancer (HR: 1.39, 95% CI: 1.04–1.87) (Table 3).

**TABLE 3:** Hazard ratios (HRs) and 95% confidence intervals (CIs) for lung cancer characteristics incidence in relation to depression at baseline (*n* = 123,961).

Characteristics	n	Age‐ adjusted model^a^	Sociodemographic‐adjusted model^b^	Fully‐adjusted models^c^
		HR (95% CI)	HR (95% CI)	HR (95% CI)
Histologic type				
Small cell lung cancer	241	1.91 (1.41–2.61)	1.63 (1.19–2.23)	1.44 (1.05–1.96)
Squamous cell carcinoma	357	1.65 (1.26–2.16)	1.36 (1.04–1.78)	1.21 (0.93–1.59)
Adenocarcinoma	1367	1.19 (1.02–1.38)	1.16 (1.00–1.35)	1.07 (0.92–1.24)
Other non‐small cell lung cancer	793	1.29 (1.06–1.56)	1.18 (0.97–1.44)	1.09 (0.90–1.33)
SEER stage				
Localized	820	1.32 (1.10–1.60)	1.24 (1.03–1.50)	1.15 (0.95–1.38)
Regional	642	1.59 (1.30–1.94)	1.49 (1.21–1.82)	1.35 (1.10–1.66)
Distant	1046	1.21 (1.02–1.44)	1.12 (0.94–1.33)	1.02 (0.86–1.21)
Unknown	244	—	—	—
Grading				
Well differentiated	299	1.57 (1.17–2.10)	1.49 (1.11–2.00)	1.39 (1.04–1.87)
Moderately differentiated	519	1.21 (0.95–1.55)	1.15 (0.90–1.47)	1.05 (0.82–1.34)
Poorly differentiate	484	1.26 (0.98–1.61)	1.13 (0.88–1.45)	1.02 (0.79–1.31)
Anaplastic	98	1.43 (0.84–2.41)	1.28 (0.75–2.18)	1.13 (0.67–1.93)
Unknown	1358	—	—	—

^a^ Model 1 adjusted for age (continuous).

^b^ Model 2 adjusted for Model 1 + education (high school or less, some college or technical training, college degree or higher), race (American Indian or Alaskan Native, Asian, Native Hawaiian/Other Pacific Islander, Black, White, More than one race, Unknown/Not reported), history of emphysema (yes/no), family history of cancer (yes/no), body mass index (< 25, 25–< 30, ≥ 30), diet quality score (continuous), physical activity (continuous), study group (CTs, or OS), HRT trial (yes/no), DM trial (yes/no), CAD trial (yes/no).

^c^ Model 3 adjusted for Model 2 + pack‐years of smoking (never smoker, < 5, 5–< 20, ≥ 20).

We also examined the association among never smokers; the result showed that, after controlling for confounding factors, there was no significant difference in the incidence of lung cancer between women with depression and those without (Table S1).

The mediation analyses results revealed that pack‐years of smoking partially mediated the depression–lung cancer relationship (Table 4). The CDE (coefficient: 0.19, 95% CI: 0.07–0.30) was the main contributor and accounted for 70.53% of the total effect. Additionally, the PIE (coefficient: 0.06, 95% CI: 0.06–0.07) contributed 24.13% of the total effect, and the overall proportion mediated (including PE and INTmed) accounted for 26.86% of the total effect. There was no evidence of a reference interaction between the exposure and the mediator (coefficient: 0.01, 95% CI: −0.01 to –0.03). Because smoking contributed 27% of the relationship, we sought potential stronger mediators such as physical activity and quality of diet. However, we only found a minimal mediating effect: for quality of diet, it accounted for only 12.15% of the total effect (Table S2). Neither physical activity played a substantial moderating effect between depression and lung cancer (Table S3).

**TABLE 4:** The effects of depression on lung cancer incidence due to mediation and interaction with pack‐years of smoking at the median value.

	Estimate	95% CI	*p*	% mediated
TE	0.26	0.14–0.39	< 0.001	100
CDE	0.19	0.07–0.30	0.002	70.53
INTref	0.01	−0.01‐0.03	0.536	2.62
INTmed	0.01	0.00–0.01	0.052	2.73
PIE	0.06	0.06–0.07	< 0.001	24.13
Proportion mediated	26.86%	0.16–0.38	< 0.001	
Proportion attributable to interaction	5.35%	−0.05‐0.15	0.298	
Proportion eliminated	29.47%	0.16–0.43	< 0.001	

*Note:* To interaction: (INTref + INTmed)/Total Effect; Proportion eliminated: (INTref + INTmed + PIE)/Total Effect. Model adjusted for age, race, education, history of emphysema, history of family cancer, body mass index, diet quality score, physical activity, study group, HRT trial, DM trial, CAD trial.

Abbreviations: CDE, controlled direct effect; INTmed, mediated interaction effect; INTref, reference interaction effect; PIE, pure indirect effect; Proportion mediated, (INTmed + PIE)/Total Effect; Proportion attributable; TE, total effect.

The results from the sensitivity analyses using 0.009 as the cut‐point of depressive symptoms aligned with the main results, both in the association between depression and incident lung cancer and causal mediation analysis (Table S4). The association remained mediated by pack‐years of smoking even when the pack‐years were at the 75th percentile (Tables S5, S6).

## 4 Discussion

We observed that depression (women with either depressive symptoms or who have been diagnosed with depression with antidepressant use) was correlated with an increased risk of developing lung cancer. Smoking partly mediated this association, accounting for about 30% of the overall effect of depression on lung cancer.

Our findings align with previous studies. The NHS study, which involved 1009 lung cancer cases among 42,913 women aged 30–55 years over a 24‐year period, discovered that higher depressive symptoms were associated with a higher risk of developing lung cancer (HR: 1.25, 95% CI: 1.04–1.51) [13]. Another prospective cohort study conducted in Taiwan included 51 cases of lung cancer among 9826 men and women and found that major depressive disorder was linked to an increased lung cancer risk (SIR: 1.65, 95% CI: 1.25–2.17) in both genders [12]. On the contrary, a study conducted by Gross et al. involving 3,177 adults of both genders in the United States over a span of 24 years found no association between depression and an elevated risk of developing lung cancer [30]. This discrepancy in findings can be explained by the fact that the study used the DIS for measuring depression, which has a relatively low specificity. This may lead to misclassification of the depression and a null finding.

The mechanism for the adverse effect of depression on lung cancer initiation can be explained by both the direct and indirect effects. The biological pathways are referred to as a direct effect on lung cancer, including factors like inflammation and an impaired immune system. Similarly, depression exhibits comparable dysregulation [31]. For instance, depression is associated with physiological changes such as decreased immune function and chronic inflammation [32]. These physiological changes may weaken the body's defenses against cancer cells and increase the likelihood of cancer.

Also, findings from mediation and moderation studies suggest the indirect pathways by which depression influences lung cancer through unhealthy behavior (e.g., smoking and quality of diet). Our study finding of smoking as a mediator is consistent with those from Trudel‐Fitzgerald et al., which demonstrated that cumulative pack‐years of smoking explained 38% of the overall depressive symptoms and lung cancer risk [13]. Individuals with depression are more inclined to use smoking as a way to alleviate emotional pain and stress [15]. Although smoking is not only a coping mechanism for depression, it may also make it harder for people with depression to quit due to the short‐term calming effects of nicotine [33]. Since smoking is a significant contributor to lung cancer, those who are depressed and simultaneously have long‐term smoking significantly increase the risk of lung cancer. Previous studies also suggested that people with depression tend to have a lower‐quality diet than the general population [34]. Poor diet quality, such as high fatty acids and low fiber, may also contribute to systemic inflammation leading to a higher incidence of lung cancer [35], highlighting its mediating role.

To the extent of our knowledge, our study is the first to explore the relationship between depression and lung cancer development focusing on specific subtypes. Our study identified a link between depression and the occurrence of small‐cell lung cancer, which highlights the significance of histologic classification in predicting the prognosis and tailoring treatment strategies for lung cancer patients. Besides, this association was observed in the well‐differentiated and regional subtypes, specifically suggesting these early and less aggressive cancer subtypes may be more strongly influenced by depression; this warrants replication and mechanistic studies with different populations.

### 4.1 Study Limitations

The strengths of our study include the substantial number of participants from the prospective cohort with a comprehensive set of covariates. However, the study has several limitations. Firstly, the CES‐D does not diagnose clinical depression but rather assesses depressive symptoms. Since it relies on self‐reported data, there is potential for recall or reporting bias. However, we also considered the antidepressant use due to the strong association between depressive symptoms and antidepressant use [36]. Secondly, data on smoking behavior over time may be influenced by recall and reporting bias, which may affect the mediating and moderating results of smoking. Thirdly, we only considered the association of baseline depression and lung cancer incidence and did not account for subsequent changes in depression. Fourthly, although several potential confounding factors have been adjusted in the models, residual confounding from unmeasured factors could affect the results. Lastly, white women made up 80%, which may restrict the ability to generalize the findings to more diverse populations.

### 4.2 Clinical Implications

Firstly, it emphasizes the importance of integrating psychological support into traditional smoking cessation programs to prevent lung cancer. Secondly, our findings call for further research to elucidate the mechanisms to explore how depression might directly increase the risk of lung cancer, independent of its role in smoking habits. Gaining insights into these direct links could pave the way for innovative treatments that address both the psychological and physical dimensions of lung cancer prevention, specifically for postmenopausal women.

## 5 Conclusion

Our results revealed a significant association between depression and the incidence of lung cancer in postmenopausal women, with pack‐years of smoking partially mediating this relationship. Moreover, the association between depression and lung cancer risk differed across various subtypes. This highlights that managing depression may play a key role in reducing lung cancer risk and decreasing tobacco use. Psychological support should be integrated with traditional smoking cessation programs for lung cancer prevention. Additionally, further research is necessary to explore the mechanisms through which depression could elevate lung cancer risk, beyond its impact on smoking behavior.

## Author Contributions

**Yuanyuan La:** conceptualization (equal), formal analysis (lead), methodology (equal), writing – original draft (lead). **Su Yon Jung:** methodology (equal), writing – review and editing (equal). **Xiaoyun Liang:** conceptualization (equal), supervision (equal), writing – review and editing (equal). **Michelle J. Naughton:** methodology (supporting), writing – review and editing (equal). **Michael Hendryx:** writing – review and editing (equal). **Juhua Luo:** conceptualization (equal), methodology (equal), writing – review and editing (equal).

## Ethics Statement

This study utilized de‐identified data from the Women's Health Initiative (WHI), a federally funded study approved by Institutional Review Boards (IRBs) and in line with the principles of the Declaration of Helsinki. The study was approved by WHI institutional review boards, and the Manuscript Proposal number is 5074.

## Conflicts of Interest

The authors declare no conflicts of interest.

## Supporting information


Data S1.

## Data Availability

The datasets of the current study are available from the corresponding author on reasonable request.
