# Supplementary material for: Association Between Depression and Lung Cancer Risk Among Postmenopausal Women
Source: Cancer Med. 2025 Mar 2;14(5):e70695. doi: 10.1002/cam4.70695 (PMC11872688; doi:10.1002/cam4.70695)
Supplement: Supplementary file 1 — Data S1. [file CAM4-14-e70695-s001.docx]

Supplementary Table 1. Hazard ratios (HRs) and 95% confidence interval (CIs) for lung cancer incidence in relation to depression among never smokers (*n*=64,905)

|  | n | Number of cases | age- adjusted model ^a^: |  | sociodemographic-adjusted model ^b^: |
| --- | --- | --- | --- | --- | --- |
|  |  |  | HR (95%CI) |  | HR (95%CI) |
| Depression |  |  |  |  |  |
| No | 56,514 | 574 | 1 |  | 1 |
| Yes | 8,391 | 68 | 0.95(0.74-1.22) |  | 0.98(0.76-1.26) |

a: Model 1 adjusted for age (continuous);

b: Model 2 adjusted for Model 1 + education (high school or less, some college or technical training, college degree or higher), race (American Indian or Alaskan Native, Asian, Native Hawaiian/Other Pacific Islander, Black, White, More than one race, Unknown/Not reported), history of emphysema (yes/no), family history of cancer (yes/no), body mass index (<25, 25-<30, ≥30), diet quality score (continuous), physical activity (continuous) , study group(CTs, OS), HRT trial (yes/no), DM trial (yes/no), CAD trial (yes/no).

Supplementary Table 2. The effects of depression on lung cancer incidence due to mediation and interaction with diet quality score at the median value

|  | Estimate | 95% CI | *P* value | % mediated |
| --- | --- | --- | --- | --- |
| TE | 0.12 | 0.04-0.21 | 0.004 | 100 |
| CDE | 0.10 | 0.02-0.19 | 0.019 | 82.80 |
| INTref | 0.01 | -0.01-0.01 | 0.082 | 5.04 |
| INTmed | 0.01 | 0.00-0.01 | 0.013 | 6.82 |
| PIE | 0.01 | 0.00-0.01 | < 0.001 | 5.34 |
| Proportion mediated | 12.15% | 0.03-0.21 | 0.009 |  |
| Proportion attributable to interaction | 11.86% | -0.01-0.25 | 0.080 |  |
| Proportion eliminated | 17.20% | 0.02-0.32 | 0.027 |  |

Abbreviations: TE=total effect; CDE = controlled direct effect; INTref = reference interaction effect; INTmed = mediated interaction effect; PIE = pure indirect effect; Proportion mediated = (INTmed + PIE) / Total Effect; Proportion attributable

to interaction: (INTref + INTmed) / Total Effect; Proportion eliminated: (INTref + INTmed + PIE) / Total Effect.

*Model adjusted for age, race, education, history of emphysema, history of family cancer, body mass index, pack years of smoking, physical activity, study group, HRT trial, DM trial, CAD trial.

Supplementary Table 3. The effects of depression on lung cancer incidence due to mediation and interaction with physical activity at the median value

|  | Estimate | 95% CI | *P* value | % mediated |
| --- | --- | --- | --- | --- |
| TE | 0.12 | 0.04-0.21 | 0.004 | 100 |
| CDE | 0.13 | 0.04-0.21 | 0.003 | 101.61 |
| INTref | 0.00 | -0.00-0.01 | 0.406 | 1.32 |
| INTmed | -0.01 | -0.01-0.00 | 0.108 | -4.87 |
| PIE | 0.00 | -0.01-0.06 | 0.240 | 1.93 |
| Proportion mediated | 2.94% | -0.08-0.03 | 0.292 |  |
| Proportion attributable to interaction | 3.55% | -0.08-0.01 | 0.107 |  |
| Proportion eliminated | 1.61% | -0.04-0.07 | 0.174 |  |

Abbreviations: TE=total effect; CDE = controlled direct effect; INTref = reference interaction effect; INTmed = mediated interaction effect; PIE = pure indirect effect; Proportion mediated = (INTmed + PIE) / Total Effect; Proportion attributable

to interaction: (INTref + INTmed) / Total Effect; Proportion eliminated: (INTref + INTmed + PIE) / Total Effect.

*Model adjusted for age, race, education, history of emphysema, history of family cancer, body mass index, pack years of smoking, quality of diet, study group, HRT trial, DM trial, CAD trial.

Supplementary Table 4. Hazard ratios (HRs) and 95% confidence interval (CIs) for lung cancer incidence in relation to depression at baseline at the cutpoint of 0.009 instead of 0.06 (*n*=123,961)

|  | n | age- adjusted model ^a^: |  | sociodemographic-adjusted model ^b^: |  | fully-adjusted models ^c^: |
| --- | --- | --- | --- | --- | --- | --- |
|  |  | HR (95%CI) |  | HR (95%CI) |  | HR (95%CI) |
| Depression |  |  |  |  |  |  |
| No | 90, 811 | 1 |  | 1 |  | 1 |
| Yes | 33,10 | 1.29(1.20-1.39) |  | 1.21(1.12-1.30) |  | 1.12(1.04-1.21) |
| Depressive symptoms |  |  |  |  |  |  |
| No | 93,825 | 1 |  | 1 |  | 1 |
| Yes | 30,136 | 1.27(1.18-1.37) |  | 1.18(1.09-1.28) |  | 1.11(1.03-1.20) |

a: Model 1 adjusted for age (continuous);

b: Model 2 adjusted for Model 1 + education (high school or less, some college or technical training, college degree or higher), race (American Indian or Alaskan Native, Asian, Native Hawaiian/Other Pacific Islander, Black, White, More than one race, Unknown/Not reported), history of emphysema (yes/no), body mass index (<25, 25-<30, ≥30), diet quality score (continuous), physical activity (continuous) , study group(CTs, OS), HRT trial (yes/no), DM trial (yes/no), CAD trial (yes/no);

c: Model 3 adjusted for Model 2 + pack-years of smoking (never smoker, <5, 5-<20, ≥20).

Supplementary Table 5. The effects of depression (cut-point: 0.009) on lung cancer incidence due to mediation and interaction with pack-years of smoking

|  | Estimate | 95% CI | *P* value | % mediated |
| --- | --- | --- | --- | --- |
| TE | 0.20 | 0.10-0.29 | < 0.001 | 100 |
| CDE | 0.13 | 0.04-0.21 | 0.005 | 63.56 |
| INTref | 0.01 | -0.01-0.03 | 0.251 | 5.13 |
| INTmed | 0.01 | 0.00-0.01 | 0.027 | 2.92 |
| PIE | 0.06 | 0.05-0.06 | < 0.001 | 28.39 |
| Proportion mediated | 31.31% | 0.18-0.45 | < 0.001 |  |
| Proportion attributable to interaction | 8.05% | -0.02-0.18 | 0.119 |  |
| Proportion eliminated | 36.44% | 0.20-0.53 | < 0.001 |  |

Abbreviations: TE=total effect; CDE = controlled direct effect; INTref = reference interaction effect; INTmed = mediated interaction effect; PIE = pure indirect effect; Proportion mediated = (INTmed + PIE) / Total Effect; Proportion attributable

to interaction: (INTref + INTmed) / Total Effect; Proportion eliminated: (INTref + INTmed + PIE) / Total Effect.

*Model adjusted for age, race, education, history of lung cancer, body mass index, diet quality score, physical activity, study group, HRT trial, DM trial, CAD trial.

Supplementary Table 6. The effects of depression on lung cancer incidence due to mediation and interaction with pack-years of smoking at the 75th percentile

|  | Estimate | 95% CI | *P* value | % mediated |
| --- | --- | --- | --- | --- |
| TE | 0.20 | 0.10-0.29 | < 0.001 | 100 |
| CDE | 0.14 | 0.04-0.23 | 0.005 | 69.14 |
| INTref | -0.01 | -0.01-0.01 | 0.882 | -0.45 |
| INTmed | 0.01 | 0.00-0.01 | 0.027 | 2.92 |
| PIE | 0.06 | 0.05-0.06 | < 0.001 | 28.39 |
| Proportion mediated | 31.31% | 0.18-0.45 | < 0.001 |  |
| Proportion attributable to interaction | 2.47% | -0.05-0.10 | 0.531 |  |
| Proportion eliminated | 30.86% | 0.15-0.46 | < 0.001 |  |

Abbreviations: TE=total effect; CDE = controlled direct effect; INTref = reference interaction effect; INTmed = mediated interaction effect; PIE = pure indirect effect; Proportion mediated = (INTmed + PIE) / Total Effect; Proportion attributable

to interaction: (INTref + INTmed) / Total Effect; Proportion eliminated: (INTref + INTmed + PIE) / Total Effect.

*Model adjusted for age, race, education, history of lung cancer, body mass index, diet quality score, physical activity, study group, HRT trial, DM trial, CAD trial.


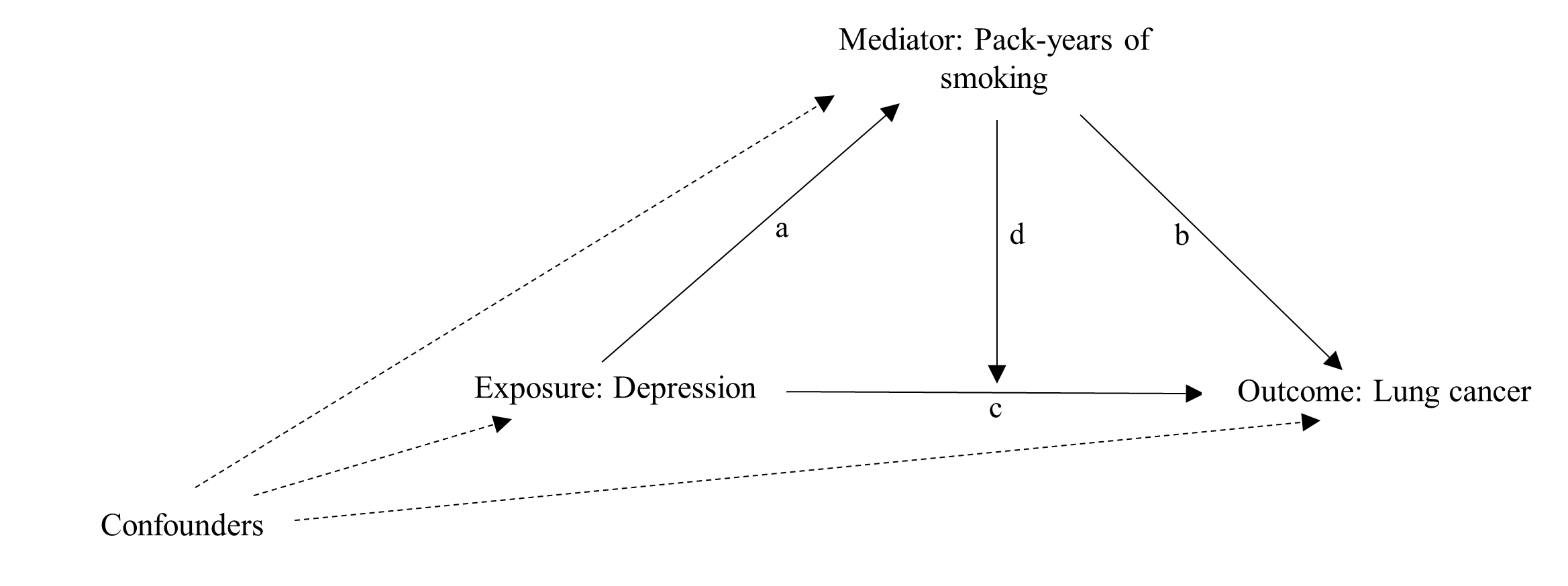


*Mediation effects are decomposed into controlled direct effects (through arrow c), reference interaction (through arrows c and d), mediated interaction (through arrows a, c, and d), and pure indirect effects (through arrows a and b).

Supplementary figure 1. Directed acyclic graph for the relationship between depression and lung cancer, and mediation through pack-years of smoking.
